# Hyperlipidemia in Saudi Arabian Patients With Systemic Lupus Erythematosus

**DOI:** 10.7759/cureus.74653

**Published:** 2024-11-28

**Authors:** Alham Alonaizan, Abdulmajed Altamimi, Hjazia Alnaqla, Mohammed S Alanazi, Naif Benragosh, Abdulrahman Alanazi, Fahad Alanazi, Lina Aldakhil, Nayef Al Ghanim

**Affiliations:** 1 Rheumatology, Internal Medicine, Security Forces Hospital Program, Riyadh, SAU; 2 Internal Medicine, King Saud Medical City, Riyadh, SAU; 3 Internal Medicine, King Saud University, Riyadh, SAU; 4 Research, King Saud Medical City, Riyadh, SAU; 5 Rheumatology, King Saud Medical City, Riyadh, SAU

**Keywords:** hydroxychloroquine, lipid profile, lupus nephritis, saudi arabia, steroids, systemic lupus erythematosus

## Abstract

Background

Systemic lupus erythematosus (SLE) is a chronic autoimmune disease associated with increased cardiovascular risk, partly due to dyslipidemia. This study aimed to evaluate the lipid profiles of Saudi Arabian patients with SLE and examine the impact of hydroxychloroquine (HCQ) and steroid use on these profiles, with a particular focus on patients with lupus nephritis.

Methods

A retrospective observational study was conducted at King Saud Medical City, Riyadh, Saudi Arabia, including SLE patients treated at the hospital’s rheumatology clinic between July 2023 and December 2023. Patients aged 15-80 years diagnosed with SLE per the American College of Rheumatology revised criteria were included. Exclusion criteria comprised menopausal or pregnant women, individuals with significant comorbid conditions, and those on specific medications. Lipid profiles, including total cholesterol, low-density lipoprotein (LDL) cholesterol, high-density lipoprotein (HDL) cholesterol, triglycerides, and very low-density lipoprotein (VLDL) cholesterol, were analyzed, and correlations with disease activity parameters were assessed using STATA software (StataCorp LLC, College Station, TX, US). Statistical analyses included Wilcoxon signed-rank tests and Spearman rank correlations.

Results

The study included 138 SLE patients (84.5% females, mean age 37.13 ± 12.9 years). Lipid profiles showed varied results: mean total cholesterol was 4.48 mmol/L, LDL 2.56 mmol/L, HDL 1.32 mmol/L, triglycerides 1.40 mmol/L, and VLDL 0.63 mmol/L. HCQ use was associated with higher, albeit not statistically significant, lipid levels. Steroid use did not show significant effects on lipid levels. Patients with lupus nephritis had higher triglyceride and VLDL levels compared to those without nephritis (p = 0.02). No significant differences were observed in lipid profiles between patients with and without anti-double-stranded DNA (dsDNA) antibodies. Significant correlations were found between triglycerides and C-reactive protein (CRP), creatinine, and erythrocyte sedimentation rate (ESR).

Conclusion

This study highlights the complex relationship between SLE, dyslipidemia, and treatment. While HCQ use did not significantly alter lipid profiles, lupus nephritis was associated with worse lipid abnormalities. These findings underscore the need for ongoing monitoring and targeted management of lipid profiles in SLE patients to mitigate cardiovascular risk.

## Introduction

Systemic lupus erythematosus (SLE) is a chronic autoimmune disease characterized by widespread inflammation that affects multiple organs, including the skin, joints, kidneys, heart, lungs, and nervous system. SLE predominantly affects women of childbearing age, particularly those between 15 and 45 years old [1]. The leading causes of mortality in SLE include infection, lupus nephritis, and atherosclerotic cardiovascular disease, with characteristic pathological features such as immune complex deposition, widespread vasculitis, and chronic inflammation contributing to organ damage and failure [2]. Patients with SLE have more than three times the risk of dying from cardiovascular disease compared to the general population, and females with SLE aged 35-44 years are 50 times more likely to develop atherosclerotic cardiovascular disease than their age-matched counterparts [3,4]. The increased risk of premature atherosclerosis in SLE is believed to result from a complex interplay of traditional and nontraditional cardiovascular risk factors [4,5]. While nontraditional factors contribute to premature atherosclerosis, traditional risk factors, particularly lipid abnormalities, remain critical therapeutic targets [4].

Multiple patterns of abnormal lipid profiles have been identified in SLE. One of the most prominent patterns, directly related to the disease, is characterized by increased serum levels of triglycerides (TG) and very low-density lipoprotein (VLDL) cholesterol and decreased high-density lipoprotein (HDL) cholesterol levels, commonly referred to as the “lupus pattern.” During active disease phases, a more pronounced increase in VLDL and TG levels, along with a decrease in HDL and low-density lipoprotein (LDL) cholesterol levels, occurs, which is described as the “active lupus pattern” [6]. Additional lipid abnormalities, such as elevated LDL cholesterol (LDL-C) and reduced HDL cholesterol (HDL-C), are frequently observed in patients with lupus nephritis or those undergoing glucocorticoid therapy, further complicating cardiovascular risk profiles in these patients.

Hydroxychloroquine (HCQ) is an antirheumatic drug widely used in the management of SLE. In addition to addressing constitutional symptoms and mild organ involvement, HCQ has demonstrated lipid-lowering properties, which can help mitigate some of the dyslipidemia associated with SLE [6,7]. Despite these benefits, abnormal lipid profiles in SLE patients are often underrecognized and undertreated. Research indicates that HCQ can reduce total cholesterol and LDL-C levels by up to 15%, though this reduction varies depending on individual patient factors and study populations [7,8]. However, HCQ alone may not be sufficient for the comprehensive management of dyslipidemia, especially when patients have significant lipid abnormalities or are undergoing glucocorticoid therapy [9].

Understanding how variations in lipid profiles relate to disease activity and contribute to premature atherosclerosis is crucial for improving patient outcomes. This study aims to investigate the lipid profiles of Saudi Arabian patients with SLE, both with and without lupus nephritis, and to assess the impact of HCQ and steroid intake on these profiles. By examining these relationships, we seek to determine how well HCQ and other treatments address dyslipidemia in SLE and to identify any gaps in current management strategies. Additionally, exploring the correlation between lipid profiles and disease activity parameters will help in prioritizing high-risk patients for targeted prevention strategies and more effective treatment plans.

## Materials and methods

Study design and population

This retrospective observational study was conducted at King Saud Medical City, Riyadh, Saudi Arabia, involving patients with SLE treated at the rheumatology clinic between July and December 2023. Patients aged 15-80 years and diagnosed with SLE according to the ACR criteria were included [10]. To ensure the accuracy of lipid profile assessments and minimize confounding factors, certain groups were excluded from the study. Specifically, pregnant women were excluded due to the significant hormonal fluctuations associated with pregnancy, which can independently affect lipid metabolism. Additionally, patients with a history of coronary artery disease, stroke, diabetes mellitus, liver disease, thyroid disease, or polycystic ovarian syndrome were excluded to avoid the influence of these conditions on lipid levels. Similarly, individuals on lipid-lowering drugs, hormonal therapy, or thyroid medications, as well as those with a history of smoking or alcohol use, were also excluded.

Data collection

Data was extracted from the hospital’s electronic records, including demographic information, duration of SLE, medication history, and laboratory tests such as erythrocyte sedimentation rate (ESR), C-reactive protein (CRP), aspartate aminotransferase (AST), alanine aminotransferase (ALT), blood urea nitrogen (BUN), and serum creatinine (Cr). Serum lipid profiles and immunological markers, including anti-double-stranded DNA (anti-dsDNA) antibodies, complement component 3 (C3), and complement component 4 (C4), were also assessed. The SLE Disease Activity Index (SLEDAI) was used to measure disease activity.

Statistical analysis

Data were analyzed using STATA (version 13, StataCorp LLC, College Station, TX, US), and lipid profile values were presented as mean (SD) and median (IQR). Non-parametric tests were employed due to non-normal distribution, with the Wilcoxon signed-rank test for variable comparisons and Spearman correlation for disease activity. A p-value of <0.05 was considered statistically significant.

## Results

Clinicodemographic characteristics

A total of 137 patients with SLE were included in the study. The majority were females (84.7%). The mean age was 37.14 ± 13 years. Arthritis was the most prevalent clinical feature (60.6%), followed by skin rash (51.8%) and lupus nephritis (44.5%). Other symptoms included hair loss (39.4%), hematological abnormalities (30.7%), serositis (21.2%), and neurological manifestations (18.2%). The average SLEDAI score was 11.42 ± 8.23, reflecting moderate disease activity.

Regarding medications, hydroxychloroquine (HCQ) was used by 88.3% of patients, with doses of 200 mg/day in 53.7% and 400 mg/day in 46.3%. The mean HCQ treatment duration was 6.98 ± 4.88 years. Corticosteroids were taken by 59.9%, primarily at doses < 5 mg/day (65.9%), with a mean duration of 4.12 ± 3.82 years. Azathioprine (40.1%) and mycophenolate mofetil (39.4%) were the most commonly used immunosuppressants, while cyclophosphamide was prescribed less frequently (7.3%). Belimumab use was rare (1.5%) (Table 1).

**Table 1:** Clinicodemographic characteristics of the study patients (N = 137) *^1^*Mean (SD), n (%) SLE: systemic lupus erythematosus; SLEDAI: Systemic Lupus Erythematosus Disease Activity Index; HCQ: hydroxychloroquine

Characteristic	N = 137^1^
Sex	
Female	116 (84.7%)
Male	21 (15.3%)
Age (years)	37.14 (13)
Clinical manifestations	
Arthritis	83 (60.6%)
Skin rash	71 (51.8%)
Lupus nephritis	61 (44.5%)
Hair loss	54 (39.4%)
Hematological	42 (30.7%)
Serositis	29 (21.2%)
Neurological	25 (18.2%)
SLEDAI score	11.42 (8.23)
Medications	
HCQ	121 (88.3%)
HCQ 200 mg/day dose	65 (53.7%)
HCQ 400 mg/day dose	56 (46.3%)
Duration of HCQ use (years)	6.98 (4.88)
Corticosteroids	82 (59.9%)
5 mg/day dose	54 (65.9%)
> 5 mg/day dose	28 (34.1%)
Duration of steroids use (years)	4.12 (3.82)
Azathioprine	55 (40.1%)
Mycophenolate mofetil	54 (39.4%)
Cyclophosphamide	10 (7.3%)
Belimumab	2 (1.5%)

Lipid profile characteristics

Table 2 presents a summary of the lipid profile among SLE patients. The total cholesterol (TC) level among the SLE patients ranged from 2.42 to 10.2 mmol/l with a median value of 4.32 (1.34) mmol/l, while the LDL levels ranged between 0.68 and 7 mmol/l with a median level of 2.43 (0.99) mmol/l.

**Table 2:** Lipid profile characteristics of the study patients (N = 137) TC: total cholesterol; LDL: low-density lipoprotein cholesterol; HDL: high-density lipoprotein cholesterol; TG: triglycerides; VLDL: very low-density lipoprotein cholesterol; SD: standard deviation; IQR: interquartile range

Lipid profile	Mean	SD	Median	IQR	Min	Max
TC (mmol/l)	4.48	1.29	4.33	1.34	2.42	10.2
LDL (mmol/l)	2.56	0.98	2.43	0.99	0.68	7
HDL (mmol/l)	1.32	0.46	1.31	0.61	0.38	2.7
TG (mmol/l)	1.40	0.83	1.16	0.83	0.25	4.45
VLDL (mmol/l)	0.63	0.38	0.53	0.39	0.11	2.02

Effect of hydroxychloroquine intake

In terms of HCQ intake, patients taking HCQs tended to have generally higher values for all lipid profile variables as compared to those who did not take HCQs; however, this difference was not statistically significant (Table 3). Patients who were using HCQs had a higher TC level of (median (IQR) = 4.33 (1.34) mmol/l) compared to 3.75 (0.88) mmol/l; similar results were seen for LDL and HDL levels in SLE patients who were taking HCQs.

**Table 3:** Lipid profile characteristics of the study patients stratified according to HCQ intake (N = 133) TC: total cholesterol; LDL: low-density lipoprotein cholesterol; HDL: high-density lipoprotein cholesterol; TG: triglycerides; VLDL: very low-density lipoprotein cholesterol; SD: standard deviation; IQR: interquartile range; HCQ: hydroxychloroquine * p-values are obtained using the Wilcoxon signed-rank test for comparisons of median lipid profile characteristics between patients with and without HCQ intake. A p-value <0.05 is considered statistically significant.

Lipid profile, median (IQR)	No HCQ (N =12)	HCQ (N = 121)	p-value*
TC (mmol/l)	3.75 (0.88)	4.33 (1.34)	0.09
LDL (mmol/l)	1.86 (0.93)	2.43 (1.00)	0.11
HDL (mmol/l)	1.09 (0.66)	1.33 (0.59)	0.36
TG (mmol/l)	1.02 (0.75)	1.16 (0.88)	0.60
VLDL (mmol/l)	0.46 (0.35)	0.53 (0.40)	0.59

Effect of steroid intake

Table 4 presents the lipid profile values for the study patients when grouped according to steroid use. There was no statistically significant difference in the lipid profile values between patients who did or did not take steroids for managing SLE. Nevertheless, TC, LDL, and HDL levels were lower among the SLE patients who took steroids. In terms of TC levels, patients who were taking steroids had a median level of 4.23 (1.38) mmol/l compared to 4.40 (1.26) mmol/l in patients who were not taking steroids. Likewise, median LDL and HDL levels were relatively lower among patients who were taking steroids. In contrast, TG and VLDL levels were higher among SLE patients taking steroids (median (IQR) = 1.19 (0.80) mmol/l and 0.54 (0.38) mmol/l, respectively).

**Table 4:** Lipid profile characteristics of study patients with SLE stratified according to steroid intake (N = 136) TC: total cholesterol; LDL: low-density lipoprotein cholesterol; HDL: high-density lipoprotein cholesterol; TG: triglycerides; VLDL: very low-density lipoprotein cholesterol; SD: standard deviation; IQR: interquartile range *p-values are obtained using the Wilcoxon signed-rank test for comparisons of median lipid profile characteristics between patients with and without steroid intake. A p-value <0.05 is considered statistically significant.

Lipid profile, median (IQR)	No steroid intake (N = 54)	Steroid intake (N = 82)	p-value*
TC (mmol/l)	4.40 (1.26)	4.23 (1.38)	0.36
LDL (mmol/l)	2.60 (1.01)	2.22 (1.04)	0.13
HDL (mmol/l)	1.35 (0.64)	1.28 (0.50)	0.54
TG (mmol/l)	1.15 (0.90)	1.19 (0.80)	0.34
VLDL (mmol/l)	0.52 (0.41)	0.54 (0.38)	0.36

Effect of lupus nephritis and anti-dsDNA antibodies

About 45% of the patients had lupus nephritis compared to 55% who did not have lupus nephritis. Table 5 presents the lipid profile of patients stratified according to the presence of lupus nephritis. A statistically significant difference was observed in the TG and VLDL levels of patients with lupus nephritis compared to those without lupus nephritis, i.e., TG and VLDL levels were higher among SLE patients with lupus nephritis compared to those without lupus nephritis (median (IQR): TG = 1.29 (1.14) mmol/l versus 1.06 (0.65) mmol/l; p = 0.02; VLDL: 0.58 (0.51) mmol/l versus 0.49 (0.28) mmol/l; p = 0.20). Even though there was no statistically significant difference, the TC, LDL, and HDL levels were generally lower among SLE patients who had lupus nephritis. As for the anti-dsDNA antibodies, there was no statistically significant difference between the lipid profile characteristics of patients with and without such antibodies (p > 0.05; Table 6).

**Table 5:** Lipid profile characteristics of study patients with SLE stratified according to the presence of lupus nephritis (N = 136) TC: total cholesterol; LDL: low-density lipoprotein cholesterol; HDL: high-density lipoprotein cholesterol; TG: triglycerides; VLDL: very low-density lipoprotein cholesterol; SD: standard deviation; IQR: interquartile range; LN: lupus nephritis *p-values are obtained using the Wilcoxon signed-rank test for median comparisons between groups. A p-value <0.05 was considered statistically significant.

Lipid profile, median (IQR)	Without LN (N = 75)	With LN (N = 61)	p-value*
TC (mmol/l)	4.33 (1.1)	4.16 (1.57)	0.66
LDL ((mmol/l)	2.50 (0.80)	2.23 (1.31)	0.58
HDL (mmol/l)	1.34 (0.60)	1.28 (0.56)	0.11
TG (mmol/l)	1.06 (0.65)	1.29 (1.14)	0.02
VLDL (mmol/l)	0.49 (0.29)	0.58 (0.51)	0.02

**Table 6:** Lipid profile characteristics of study patients with SLE stratified according to the presence of anti-dsDNA antibodies (N = 136) TC: total cholesterol; LDL: low-density lipoprotein cholesterol; HDL: high-density lipoprotein cholesterol; TG: triglycerides; VLDL: very low-density lipoprotein cholesterol; SD: standard deviation; IQR: interquartile range; dsDNA: anti-double-stranded DNA antibodies *p-values are derived from the Wilcoxon signed-rank test for median comparisons between groups. A p-value <0.05 was considered statistically significant.

Lipid profile, median (IQR)	Without dsDNA (N = 41)	With dsDNA (N = 95)	p-value*
TC (mmol/l)	4.37 (0.90)	4.17 (1.45)	0.13
LDL (mmol/l)	2.30 (0.91)	2.43 (1.06)	0.81
HDL (mmol/l)	1.40 (0.55)	1.25 (0.61)	0.16
TG (mmol/l)	1.16 (0.73)	1.16 (0.88)	0.55
VLDL (mmol/l)	0.53 (0.34)	0.53 (0.40)	0.53

Lipid profile and systemic lupus erythematosus activity parameters

Table 7 presents a summary of the correlation analysis between different lipid profile characteristics and parameters of disease activity. The analysis revealed a statistically significant and mildly positive correlation between total cholesterol (TC) and Cr (r = 0.19; p = 0.035) and C3 (r = 0.21; p = 0.021). HDL levels were significantly positively correlated with C3 (r = 0.30; p < 0.001). Triglycerides (TG) showed significant positive correlations with Cr (r = 0.28; p = 0.002), ESR (r = 0.24; p = 0.009), and CRP (r = 0.44; p < 0.001). VLDL demonstrated weak positive linear associations with Cr (r = 0.27; p = 0.003) and ESR (r = 0.24; p = 0.007) and a moderate positive correlation with CRP levels (r = 0.44; p < 0.001). SLEDAI scores did not show a significant correlation with any lipid profile variables (r values ranging from −0.059 to 0.113 with p-values above 0.2).

**Table 7:** Results of correlation analysis between variables of disease activity and lipid profile characteristics among the study patients with SLE (N = 137) SLEDAI: Systemic Lupus Erythematosus Disease Activity Index; Cr: creatinine; C3: complement component 3; C4: complement component 4; ESR: erythrocyte sedimentation rate; CRP: C-reactive protein; 24 hr urine protein: 24-hour urine protein; TC: total cholesterol; LDL: low-density lipoprotein; HDL: high-density lipoprotein; TG: triglycerides; VLDL: very low-density lipoprotein *Correlation coefficients (r) and p-values are reported. Statistical testing was performed using Spearman's rank correlation for non-normally distributed data. A p-value of <0.05 was considered statistically significant.

Lipid profile	SLEDAI (r, p)	Cr (r, p)	C3 (r, p)	C4 (r, p)	ESR (r, p)	CRP (r, p)	24 hrs urine protein (r, p)*
TC (mmol/l)	−0.07 (0.45)	0.19 (0.035)	0.21 (0.021)	0.11 (0.22)	−0.02 (0.81)	0.00 (0.97)	0.18 (0.05)
LDL (mmol/l)	0.02 (0.86)	0.14 (0.12)	0.07 (0.45)	0.07 (0.45)	−0.07 (0.48)	−0.04 (0.67)	0.18 (0.05)
HDL (mmol/l)	−0.06 (0.52)	−0.07 (0.42)	0.30 (<0.001)	0.15 (0.11)	−0.04 (0.66)	−0.12 (0.18)	−0.08 (0.36)
TG (mmol/l)	0.11 (0.22)	0.28 (0.002)	−0.10 (0.27)	−0.08 (0.36)	0.24 (0.009)	0.44 (<0.001)	0.16 (0.07)
VLDL (mmol/l)	0.12 (0.20)	0.27 (0.003)	−0.10 (0.26)	−0.09 (0.34)	0.24 (0.007)	0.44 (<0.001)	0.16 (0.07)

## Discussion

The results of this study provide valuable insights into the lipid profile characteristics of patients with SLE and its association with various disease- and patient-related factors. The analysis revealed some interesting findings that can contribute to our understanding of SLE and its management. The TC levels of the study patients ranged from 2.42 to 10.2 mmol/L, with a mean of 4.47 mmol/L. The LDL levels ranged from 0.68 to 7 mmol/L, with a mean of 2.25 mmol/L. These findings concur with the TC and LDL levels reported by Durcan et al. (2017) for a large cohort of SLE patients [11]. In a Brazilian study involving 69 SLE female patients with dyslipidemia, hypercholesterolemia, and hypertriglyceridemia were also found [12]. These findings are consistent with previous literature reporting a high frequency of dyslipidemia in SLE patients [13,14].

Recent guidelines recommend regular lipid monitoring and management in patients with SLE, particularly given their elevated cardiovascular risk. Clinicians should assess lipid profiles at diagnosis and periodically thereafter, especially in patients with additional risk factors such as lupus nephritis or those on glucocorticoid therapy. Current management strategies advocate for lifestyle modifications, including dietary changes and increased physical activity, alongside pharmacological interventions when necessary. Statins are often recommended for SLE patients with dyslipidemia, as they have been shown to effectively lower LDL cholesterol levels and reduce cardiovascular events. HCQ remains a cornerstone of treatment for improving lipid profiles, although its effects can vary among individuals [8,14].

One of the key findings of our study is the association between HCQ intake and a disrupted lipid profile of SLE patients. We observed that SLE patients who were taking HCQs had higher levels of TC, LDL, and HDL compared to those who did not take HCQ. This is especially relevant since HCQ is commonly used in the treatment of SLE, and its potential impact on lipid metabolism and cardiovascular risk has been a topic of interest. In contrast, Petri et al. (2019) found that HCQ use was associated with lower levels of TC, LDL, and TG in SLE patients [8]. These differences could be attributed to several factors. Firstly, the study by Petri et al. might have included a different demographic of SLE patients, such as those with varying disease severity or different comorbid conditions. For instance, their cohort might have had a higher proportion of patients with milder forms of SLE or different baseline characteristics, which could influence the lipid profile outcomes. Secondly, variations in study design and methodology can significantly impact results. For example, differences in how HCQ use was measured (e.g., duration of use, dosage, or adherence rates) or variations in the timing of lipid measurements relative to HCQ initiation could lead to different findings. Additionally, the methodology for analyzing lipid profiles, such as the specific assays or criteria used for categorization, could affect results. Lastly, other confounding factors that could influence the results include variations in lifestyle factors (such as diet and physical activity), concomitant use of other medications, or genetic predispositions that affect lipid metabolism. For instance, if the population studied by Petri et al. had a higher use of statins or other lipid-altering medications, this could contribute to the observed lower lipid levels. In summary, while our findings suggest that HCQ may be associated with higher lipid levels, the study by Petri et al. shows an opposite trend. The discrepancies between these studies highlight the need to consider specific patient characteristics, methodological differences, and potential confounding factors when interpreting results.

In contrast to HCQ use, steroid intake was seen to be associated with lower levels of TC and LDL but higher levels of TGs and VLDL in patients with SLE. Steroids, particularly corticosteroids, are known to influence lipid metabolism adversely. They can induce insulin resistance, leading to increased lipolysis and elevated triglyceride levels [15]. Additionally, steroids can impair the synthesis and function of LDL receptors, contributing to the observed reduction in LDL levels while simultaneously increasing VLDL levels. This disruption in lipid metabolism is consistent with the findings of a previous study by Liang et al., which reported that long-term steroid use in SLE patients is associated with dyslipidemia and an increased risk of cardiovascular disease [16]. The present study further corroborates these results and underscores the importance of monitoring lipid profiles in SLE patients undergoing steroid therapy to mitigate potential cardiovascular risks.

Similarly, SLE patients who developed lupus nephritis had higher levels of TGs and VLDL compared to those without lupus nephritis, while TC, LDL, and HDL levels were lower in SLE patients with lupus nephritis. Lupus nephritis is a severe complication of SLE that affects the kidneys and is characterized by inflammation and damage to the renal tissue [17]. This condition can increase the risk of cardiovascular disease due to several factors, including the effect of kidney damage on lipid metabolism. Patients with lupus nephritis often experience altered lipid profiles due to the kidney's reduced ability to clear lipids from the blood. Tektonidou et al. also reported an increased prevalence of dyslipidemia in SLE patients with lupus nephritis [18]. Dyslipidemia in lupus nephritis patients is linked to both the inflammatory process associated with the condition and the metabolic changes induced by renal impairment [17,19]. Therefore, understanding the lipid profile in lupus nephritis patients is crucial for the management of cardiovascular risk in this high-risk population.

Lastly, we examined the association between different disease activity parameters and lipid profile characteristics of SLE patients. The results revealed significant correlations between lipid parameters and disease activity markers, including C3, CR, ESR, and CRP. These findings are consistent with a previous study that reported a positive association between disease activity and dyslipidemia in SLE patients [20]. These associations highlight the importance of monitoring lipid profiles as a part of the comprehensive management of SLE, especially in patients with active disease. However, it is notable that SLEDAI did not show significant correlations with lipid parameters in our study. Conversely, Yuan et al. (2016) found significant positive correlations between TG and TC levels and SLEDAI score [21]. This contradiction may reflect the complex and multifaceted nature of SLEDAI as a measure of disease activity, which incorporates various clinical and laboratory parameters beyond just lipid levels.

Study strengths

This study's strengths include its detailed patient demographics and comprehensive data collection. The diverse cohort of SLE patients from King Saud Medical City enhances the generalizability of the findings within the Riyadh region. The thorough review of medical records, covering a wide range of variables such as disease activity markers and medication history, contributes to a robust analysis of lipid profiles. Additionally, the study's exclusion of potential confounding factors like diabetes, thyroid disorders, and lipid-altering medications strengthens the validity of the results related to SLE-specific lipid abnormalities. The use of validated tools, such as SLEDAI, provides a standardized measure for comparing disease severity and its impact on lipid profiles, while the application of nonparametric tests and Spearman rank correlations offers accurate results given the data characteristics.

Study limitations

The study involved a relatively small sample size. Its retrospective design introduces potential biases and relies on existing records, which might result in incomplete data or historical management changes not being captured. The cross-sectional nature of the study provides only a snapshot of lipid profiles at one point in time, limiting the ability to assess temporal changes or establish causality between lipid abnormalities and disease activity. The absence of a control group of healthy individuals or patients with other conditions restricts the ability to determine whether observed lipid abnormalities are specific to SLE. Additionally, the variability in HCQ and steroid use was not fully accounted for, which could impact lipid profiles. The findings may also have limited generalizability beyond the specific population of SLE patients in Riyadh or Saudi Arabia, as regional differences in genetics, lifestyle, or healthcare practices might affect lipid profiles. Despite efforts to control confounding factors, residual confounding is available due to unmeasured variables that could still influence the results.

Recommendations

Based on the findings of this study, several recommendations can be made. Firstly, further research utilizing a longitudinal design is recommended to track changes in lipid profiles over time and to better understand the causal relationships between lipid abnormalities and SLE disease activity. Including a control group of healthy individuals or patients with other autoimmune conditions could provide a comparative baseline to evaluate the specificity of lipid abnormalities in SLE. It is also advisable to conduct studies with larger and more diverse cohorts to enhance the generalizability of the findings to other populations. Additionally, future research should consider a more detailed analysis of the effects of different HCQ and steroid dosages on lipid profiles, as this could offer insights into optimizing treatment strategies to mitigate adverse lipid-related effects. Implementing standardized protocols for lipid monitoring in SLE patients could improve the consistency of data and support more targeted interventions. Finally, public health initiatives aimed at educating SLE patients about cardiovascular risk factors and lipid management should be considered to improve patient outcomes and prevent cardiovascular complications associated with SLE.

## Conclusions

This study reveals significant lipid abnormalities in Saudi Arabian SLE patients, particularly those with lupus nephritis, who had higher triglyceride and VLDL levels. Hydroxychloroquine did not notably influence lipid profiles, while steroid use was linked to adverse changes, specifically increased triglycerides. These findings highlight the need for vigilant cardiovascular risk assessment in SLE patients, especially those with lupus nephritis. The study emphasizes the importance of individualized treatment plans, and further research is essential to better understand the underlying mechanisms and improve patient outcomes.
